# Eleven metabolism‑related genes composed of Stard5 predict prognosis and contribute to EMT phenotype in HCC

**DOI:** 10.1186/s12935-023-03097-0

**Published:** 2023-11-17

**Authors:** Dongping Li, Xiahui Lin, Jiale Li, Xinyi Liu, Feng Zhang, Wenqing Tang, Si Zhang, Ling Dong, Ruyi Xue

**Affiliations:** 1 Department of Gastroenterology and Hepatology, Shanghai Institute of Liver Diseases, Zhongshan Hospital, Fudan University, 180 Fenglin Road, Shanghai, 200032 China; 2 Key Laboratory of Glycoconjugate Research Ministry of Public Health, Department of Biochemistry and Molecular Biology, School of Basic Medical Sciences, Fudan University, Shanghai, 200032 China

**Keywords:** HCC, EMT, Metabolic reprogramming, Prognostic model, Stard5

## Abstract

**Background:**

Hepatocellular carcinoma (HCC) is one of the most common cancers worldwide, with a high mortality and poor survival rate. Abnormal tumor metabolism is considered a hallmark of HCC and is a potential therapeutic target. This study aimed to identify metabolism-related biomarkers to evaluate the prognosis of patients with HCC.

**Method:**

The Cancer Genome Atlas (TCGA) database was used to explore differential metabolic pathways based on high and low epithelial-mesenchymal transition (EMT) groupings. Genes in differential metabolic pathways were obtained for HCC metabolism-related molecular subtype analysis. Differentially expressed genes (DEGs) from the three subtypes were subjected to Lasso Cox regression analysis to construct prognostic risk models. Stard5 expression in HCC patients was detected by western blot and immunohistochemistry (IHC), and the role of Stard5 in the metastasis of HCC was investigated by cytological experiments.

**Results:**

Unsupervised clustering analysis based on metabolism-related genes revealed three subtypes in HCC with differential prognosis. A risk prognostic model was constructed based on 11 genes (*STARD5, FTCD, SCN4A, ADH4, CFHR3, CYP2C9, CCL14, GADD45G, SOX11, SCIN*, and *SLC2A1*) obtained by LASSO Cox regression analysis of the three subtypes of DEGs. We validated that the model had a good predictive power. In addition, we found that the high-risk group had a poor prognosis, higher proportion of Tregs, and responded poorly to chemotherapy. We also found that Stard5 expression was markedly decreased in HCC tissues, which was associated with poor prognosis and EMT. Knockdown of Stard5 contributed to the invasion and migration of HCC cells. Overexpression of Stard5 inhibited EMT in HCC cells.

**Conclusion:**

We developed a new model based on 11 metabolism-related genes, which predicted the prognosis and response to chemotherapy or immunotherapy for HCC. Notably, we demonstrated for the first time that Stard5 acted as a tumor suppressor by inhibiting metastasis in HCC.

**Supplementary Information:**

The online version contains supplementary material available at 10.1186/s12935-023-03097-0.

## Introduction

Hepatocellular carcinoma (HCC) is one of the most common malignant tumors and is the third leading cause of cancer-related deaths worldwide [1]. The 5 year survival rate is less than 12%. Most HCC cases are at advanced stages when diagnosed [2], thereby leading to poor prognosis and posing challenge to treatment. Traditionally, clinical staging and vascular tumor invasion are essential contributors to clinical outcomes and may help to predict survival [3]. However, these clinicopathological risk factors are limited in terms of prognostic evaluation and are insufficient to distinguish between high-risk and low-risk patients. Sensitivity to adjuvant chemotherapy is even more unpredictable. Thus, there is an urgent need to explore novel prognosis-related genes, building a comprehensive model to predict clinical outcomes.

Epithelial-mesenchymal transition (EMT) is a favorable feature of malignant cells [4]. Some cells lose their epithelial characteristics and obtain a mesenchymal phenotype during the transition, eventually leading to a loss of intercellular junctions [4]. Thus, EMT not only promotes invasion and metastasis, but also leads to enhanced stemness of tumor cells, contributing to the development of chemoresistance [5], immunosuppression [6] and targeted therapy resistance [7]. Therefore, developing new therapeutic strategies to control EMT is essential in oncogenesis, metastasis, and treatment. Unfortunately, reversing EMT in tumor cells has not yet been achieved [8].

Metabolic reprogramming is generally recognized to be a new hallmark of cancer [4], most notably the “Warburg effect.” In addition to dysregulated glucose metabolism, metabolic reprogramming in tumor cells is characterized by abnormal nucleotide metabolism, amino acid metabolism, mitochondrial biosynthesis, and the rest of pathways [9]. The study of these metabolic reprogramming will shed light on the molecular events of malignancy and facilitate to identify preferable approaches for diagnosis and treatment. Recent findings suggested that metabolic demand is altered in EMT-activated cells to meet increased motility and aggressiveness [10]. In some cases, metabolic reprogramming can also drive EMT, and the link between the two is reciprocal. In certain cancer types, tumors undergoing metabolic reprogramming are correlated with worse survival [11]. Metabolic reprogramming of cancer cells has tremendous impact on immune microenvironment [12], thereby influencing the efficacy of immunotherapy. Therefore, understanding the mechanisms of metabolic reprogramming in different EMT states is crucial for improving patient survival.

In this study, The Cancer Genome Atlas (TCGA)-LICH was used to analyze differential metabolic pathways according to different EMT status groups. Based on the unsupervised cluster analysis of the differential metabolic pathway genes, three clusters with significant differences in survival were obtained. Using differential expression analysis and LASSO-Cox regression, 11 genes were selected to establish a prognostic risk model. This prognostic model may help to optimize risk stratification and identify appropriate therapeutic strategies for HCC patients. Moreover, the correlation between Stard5 and EMT has been broadly verified in vitro and in patients, which provides a target for exploring the interaction between EMT and metabolic reprogramming.

## Materials and methods

### Data acquisition

The mRNA expression profiles of HCC patients and the corresponding clinical profiles, including age, gender, grade, stage, alcohol consumption, Hepatitis B, Hepatitis C and survival time, were downloaded from the TCGA-LIHC database (https://gdc.nci.nih.gov/) and were detailed in Table. 1. Validation dataset GSE14520 was downloaded from Gene Expression Omnibus (GEO) database, and survival information for the samples was shown in Table. 2.

**Table 1:** The clinical characteristics of TCGA-LIHC samples

	TCGA-tumor
Age	
> 65	138
< = 65	229
Negative	40
Gender	
MALE	248
FEMALE	119
Grade	
G1	55
G2	176
G3	119
G4	12
NA	5
Stage	
Stage I	171
Stage II	85
Stage III	83
Stage IV	4
[Discrepancy]	2
NA	22
Alcohol consumption	
Yes	115
No	252
Hepatitis B	
Yes	103
No	264
Hepatitis C	
Yes	56
No	311
EMT group	
EMT-H	19
EMT-L	348
Cluster	
Cluster1	251
Cluster2	104
Cluster3	12
OS	
Alive	237
Dead	130

**Table 2:** Survival information for GSE14520 data set

	GSE14520
OS	
Alive	136
Dead	85

### Calculation of EMT enrichment scores

A total of 145 epithelium (EPI) genes and 170 mesenchyme (MES) genes were obtained from PMID: 25214461 [13]. Based on the above gene sets, the samples’ EPI enrichment score and MES enrichment score were calculated by the R package GSVA (v1.34.0), and the EMT enrichment score was subtracted from the two. Surv_cutpoint of R package survminer (v0.4.8) was used to find the most appropriate node to differentiate EMT-H from EMT-L groups. Survival analysis of the two groups was performed by the R package surv (v3.2–7). Differences in clinical characteristics between the EMT subgroups were detected using Kruskal wallis test.

### Calculation of metabolic signature enrichment scores

Enrichment scores for metabolic pathways were calculated for samples based on metabolic pathway-related genes provided by PMID: 33917859 [14]. The differences of metabolic enrichment scores between EMT groups were analyzed using Kruskal wallis test.

### Subtypes identification based on metabolic reprogramming

A subtype analysis related to metabolic reprogramming of HCC was performed based on genes of metabolic pathways, which had significant differences between the EMT-H and EMT-L groups. Unsupervised cluster analysis was applied to all samples in the TCGA-LIHC dataset through the R package ConsensusClusterPlus (v1.50.0) with the algorithm K-means. The clusters were then analyzed for survival using R package survival and survminer.

### Functional enrichment analysis

Differentially expressed genes (DEGs) of the clusters were acquired by the R package limma [15]. A threshold of |${\mathrm{log}}_{2}foldchange$|> 1 and adjusted *p* < 0.05, were considered for DEGs. Overlapping DEGs from the three clusters were used for subsequent analysis. The Kyoto Encyclopedia of Genes and Genomes (KEGG) enrichment analysis and Gene Ontology term (GO) analysis, which consists of biological processes (BP), cellular component (CC), and molecular function (MF), were performed using DEGs shared by the three clusters [16].

### Construction of a prognostic model

Univariate Cox regression analysis was applied to the significant DEGs, using *p* < 0.01 as the threshold, in combination with the overall survival data. DEGs were then further filtered by LASSO-Cox regression analysis, and risk score models were constructed, a process that resorted to the R package glmnet (v4.0–2). Lambda screening was used for cross-validation. The model corresponding to lambda.min was used to collect the gene expression matrix. The risk score for each sample was calculated using the following equation: ${\mathbf{R}\mathbf{S}\mathbf{c}\mathbf{o}\mathbf{r}\mathbf{e}}_{{\varvec{i}}}= \sum_{{\varvec{j}}=1}^{{\varvec{n}}}{\mathbf{exp}}_{{\varvec{ji}}}\times {{\varvec{\upbeta}}}_{{\varvec{j}}}$. The median risk score was used to classify high-and low-risk groups. A *p*-value of Kaplan–Meier survival analysis < 0.05 was considered to indicate a significant difference between the two groups. The area under the curve (AUC) values for the model were calculated using the survival data and demonstrated by time-dependent receiver operating characteristic (ROC) curves, with AUC values greater than 0.6 indicating good predictive power of the prediction model.

### Immune cell infiltration and chemotherapy resistance prediction analysis

To explore the response of patients to Erlotinib, Shikonin, Metformin, Bortezomib, Metformin, and Lapatinib, the predictive value of IC50 was obtained using the R package pRRophetic (v 0.5) analysis. The difference in IC50 between the high-and low-risk groups was tested using the Wilcoxon test. R package CIBERSORT (v1.03) was used to analyze the proportion of immune cells in all patients.

### Clinical HCC patient samples and cell culture

To validate the expression levels of genes in the prognostic model, we collected 80 tumors and adjacent normal tissues from patients with HCC. All patients participating in this study signed an informed consent form. This project was approved by the Human Research Ethics Committee of Zhongshan Hospital, Fudan University (Y2021-242). Tumors and adjacent normal tissues were then collected from patients who underwent surgical resection of the liver. All tissues were obtained immediately after surgical resection and frozen at − 80 °C. Huh7 cells, derived from the Chinese Academy of Sciences, were cultured in DMEM medium (D5796, sigma) containing 10% fetal bovine serum (16140071, Gibco) at 37 °C and 5% CO_2_.

### Quantitative real-time PCR analysis

Total RNA was extracted from the tumors and adjacent normal tissues and reverse transcribed to cDNA using the Kit (EZBioscience, MN, USA). Then, quantitative PCR amplification was operated by a CFX384 real-time PCR machine (Bio-Rad, USA) using SYBR Green (Vazyme, China). Gene abundances were normalized to GAPDH. The primer sequences were shown in Table 3.

**Table 3:** Primer sequences for Real-time PCR

Gene	Primer sequences
STARD5-F	CCGGGAAGGCAATGGAGTTT
STARD5-R	TCATCCCACTTCACTCGTAGG
FTCD-F	TCCCGACTTATCGACATGAGC
FTCD-R	GCCGTACAGGTAAACTGGC
SCN4A-F	TTCACAGGGATCTACACCTTTGA
SCN4A-R	CACAAACTCTGTCAGGTACGC
ADH4-F	AGTTCGCATTCAGATCATTGCT
ADH4-R	CTGGCCCAATACTTTCCACAA
CFHR3-F	TACCAATGCCAGTCCTACTATGA
CFHR3-R	CCGACCACTCTCCATTACTACA
CYP2C9-F	GCCTGAAACCCATAGTGGTG
CYP2C9-R	GGGGCTGCTCAAAATCTTGATG
CCL14-F	CCAAGCCCGGAATTGTCTTCA
CCL14-R	GGGTTGGTACAGACGGAATGG
GADD45G-F	CAGATCCATTTTACGCTGATCCA
GADD45G-R	TCCTCGCAAAACAGGCTGAG
SOX11-F	AGCAAGAAATGCGGCAAGC
SOX11-R	ATCCAGAAACACGCACTTGAC
SCIN-F	ATGGCTTCGGGAAAGTTTATGT
SCIN-R	CATCCACCATATTGTGCTGGG
SLC2A1-F	ATTGGCTCCGGTATCGTCAAC
SLC2A1-R	GCTCAGATAGGACATCCAGGGTA

### Immunohistochemistry (IHC)

HCC and adjacent normal tissues were deparaffinized, rehydrated, blocked for endogenous peroxidase activity, antigen repair, and blocking, before being incubated overnight at 4 °C with primary antibodies against Stard5(ab178688, Abcam), N-cadherin, vimentin, E-cadherin and zo-1(9782 T, CST). The sections were then incubated with horseradish peroxidase-conjugated secondary antibodies for 1 h at room temperature and stained with 3, 3-diaminobenzidine tetrahydrochloride (DAB). Finally, cells were observed under a microscope.

### Construction of stable cell lines

The cDNA or shRNA (Genepharma, China) targeting Stard5 were recombined into lentiviral vectors to overexpress or knockdown Stard5, then transfected into 293 T cells. The mature infectious lentivirus was collected after 72 h. Stable Stard5-overexpressing and Stard5-knockdown Huh7 cell lines were constructed and verified by western blot.

### Western blot

Cells were lysed to extract total protein and heated to 100 °C for 20 min. Protein was added onto 8–12% SDS-PAGE electrophoreses and transferred to the PVDF membrane, then the blocked PVDF membrane was incubated with 1:1000 diluted Stard5 (ab178688, Abcam), β-Actin (3700 T, CST), N-cadherin, vimentin, E-cadherin and zo-1 (9782 T, CST) antibody at 4 °C overnight. After washing with TBST, the PVDF membrane was incubated with a 1:10000 diluted secondary antibody for 1.5 h at room temperature. Finally, a chemiluminescence analysis was performed.

### Wound healing assay

The cells were inoculated in six-well plates at 1 × 10^6^ cells per well to form a dense monolayer after 12 h. Lines were drawn with a 200 μL tip to the cell layer to form straight cell wounds. After washing with PBS, the cells were incubated with serum-free medium at 37 °C for 48 h. The wound width was recorded at 0 and 48 h.

### Transwell migration and invasion assays

80 μl BD Matrigel mixture (diluted 1:10 with DMEM) was pre-coated in a transwell chamber (3513, Corning) at 37 °C overnight. Cells were diluted with serum-free DMEM and 4 × 10^4^ cells were added to the upper chamber. Then, 500 μl of DMEM containing 30% fetal bovine serum was added to the bottom chamber. After incubation at 37 °C for 48 h, the chambers were fixed in 4% paraformaldehyde for 2 h. Cells in the upper chamber were removed, then stained with crystal violet, washed with PBS and photographed under a microscope.

### Statistical analysis

R software (version 4.0.1) and GraphPad Prism (version 9.0) were used for statistical analysis of the experimental data. Pair or unpaired Student t-tests were used for comparison of data between two groups. The Mann–Whitney test was used when the data did not conform to a normal distribution. One-way analysis of variance (ANOVA) was used to compare three or more groups. Differences between the groups were considered statistically significant at *p* < 0.05.

## Results

### Cluster 1 has the best prognosis based on subtypes identification of differential metabolic pathways between EMT subgroups

**Fig. 1:**
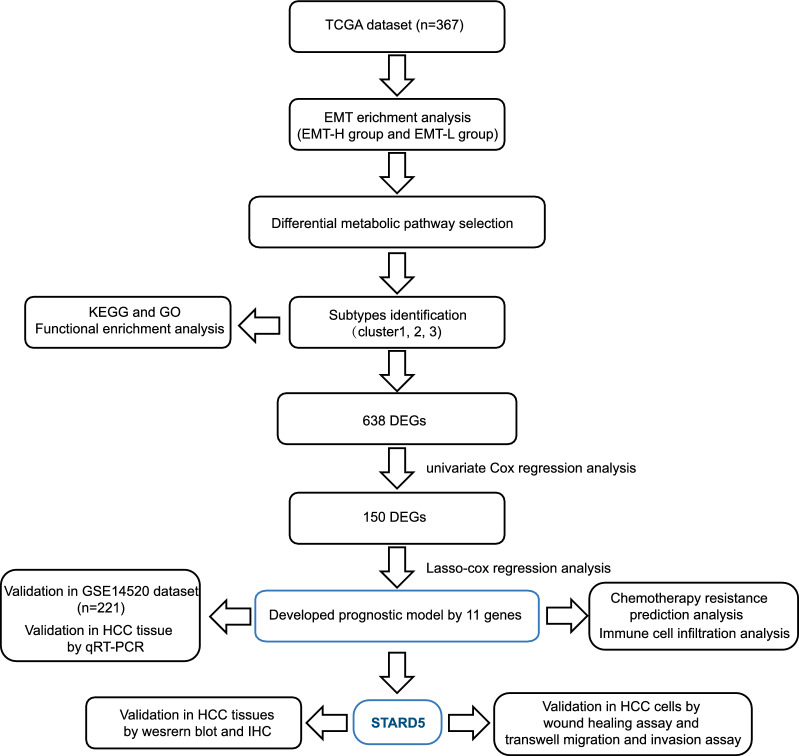
Flowchart illustrating the process of establishing a prognostic model for HCC in this study

**Fig. 2:**
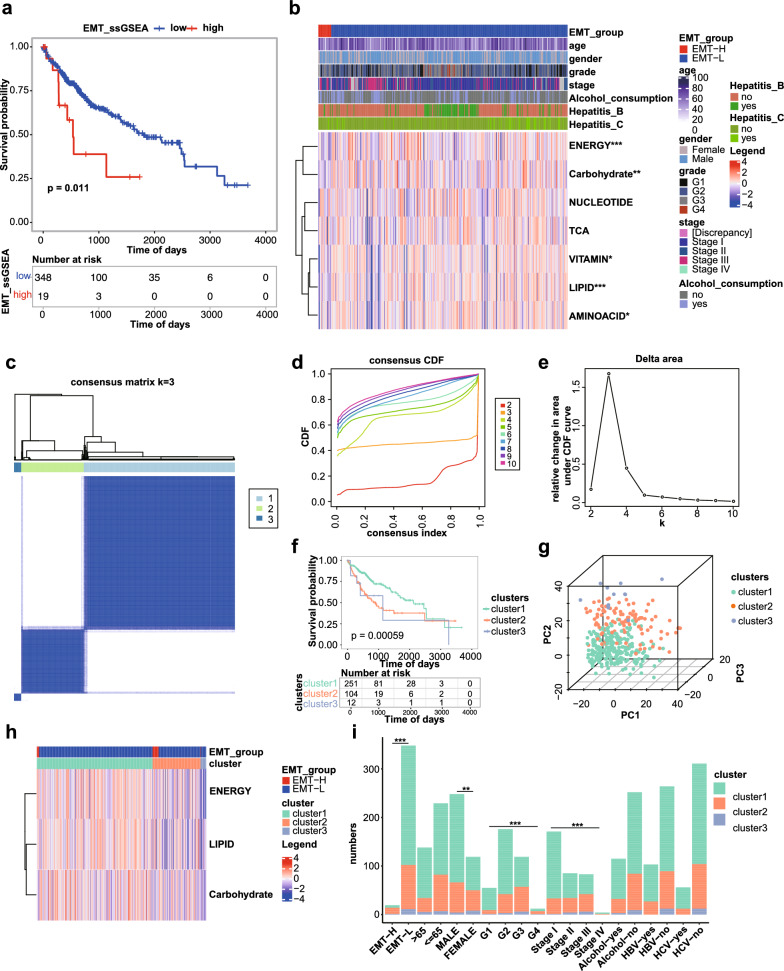
Subtype identification based on differential metabolic pathways between EMT subgroups. **a**. KM curves for EMT subgroup. **b**. Heat map of enrichment scores for seven metabolic pathways. **c**. Heat map of unsupervised cluster analysis of patients. **d**. Cumulative distribution profile. **e**. Unsupervised cluster fragmentation. **f**. KM curves for survival analysis of each subtype. **g**. PCA results based on different subtypes. **h**. Heat map of enrichment scores for differential metabolic pathways. **i**. Distribution of three subtypes in subgroups with different clinical characteristics. * p < 0.05, ** *p* < 0.01, and *** *p* < 0.001

The study flowchart was shown in Fig. 1. First, we downloaded the expression data and clinical data of LIHC from the UCSC Xena database, removing the cases with missing survival information, and finally included 367 cancer samples. Expression matrix, which contains 16515 protein-coding genes, was subsequently used in the analysis. Based on the EMT score of each sample, we obtained 348 EMT-L samples and 19 EMT-H samples. Kaplan–Meier survival analysis showed that the overall survival (OS) of the EMT-L group was significantly longer than that of the EMT-H group (Fig. 2a). We then compared the clinical characteristics between the EMT subgroups. No significant differences in clinical characteristics were observed, possibly due to the small sample size of EMT-H samples (Additional file 1: Fig. S1a). Next, we obtained the genes of LIPID, NUCLEOTIDE, Carbohydrate, TCA, ENERGY, VITAMIN, and AMINOACID metabolic pathways and calculated the pathway enrichment scores for each sample. We found that LIPID, ENERGY, Carbohydrate, VITAMIN, and AMINOACID levels were significantly different between the EMT subgroups (Fig. 2b). It is implied that metabolic reprogramming occurred when tumors underwent EMT. The shared signature genes in the different metabolic pathways were also shown, with LIPID and VITAMIN sharing the most signature genes [26] (Additional file 1: Fig. S1b). Then, unsupervised cluster analysis based on the genes contained in these five metabolic pathways was performed (Fig. 2c). The most appropriate number of cluster was three (Fig. 2d, e). The Kaplan–Meier curves showed significant differences in survival among the three subtypes (Fig. 2f). Heat maps showed that enrichment scores for three critical metabolic pathways were also different among the three subtypes (Fig. 2h). We further investigated the differences in age, gender, grade, stage, alcohol consumption, Hepatitis B, Hepatitis C, and EMT subgroups among the three clusters, and found that the grade, stage, and EMT groups differed significantly (Fig. 2i).

### Functional enrichment analysis

**Fig. 3:**
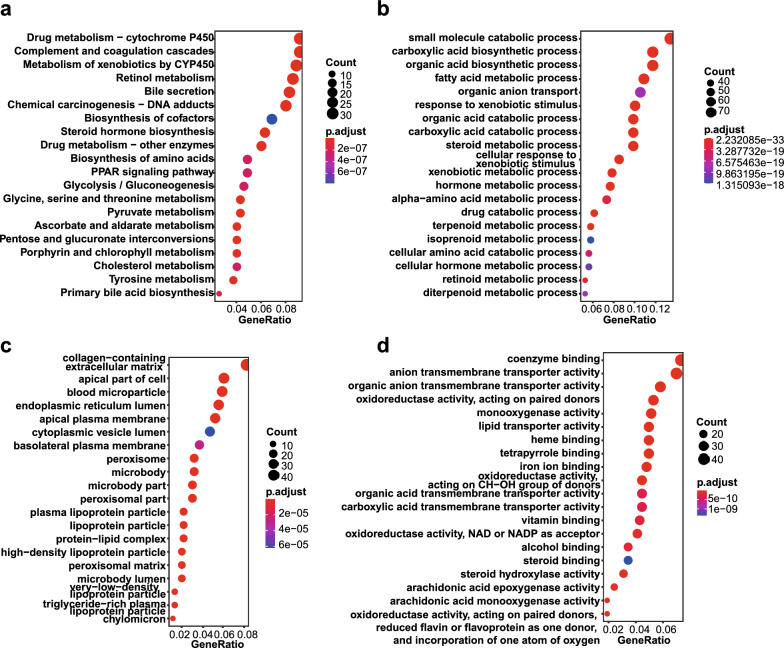
Functional enrichment analysis of differentially expressed genes (DEGs). **a** KEGG pathway enrichment analysis results, showing only the first 20 pathways. **b**–**d** GO enrichment analysis results, showing only the first 20 terms. (**b**) molecular function, (**c**) biological process and (**d**) cellular components

We retrieved the DEGs among clusters, including 1739 DEGs between cluster1 and cluster2, 4779 DGEs between cluster1 and cluster3, 2519 DEGs between cluster2 and cluster3, and finally obtained 638 DEGs after intersection of the three clusters (Additional file 2: Fig. S2a). Functional enrichment analysis was then performed on the 638 DEGs. The pathways enriched by KEGG and BP were mainly focused on metabolic pathways related to catabolic or biosynthetic process (Fig. 3a, b). The pathways enriched for CC mainly focused on cell adhesion, such as collagen-containing extracellular matrix, apical part of cell, etc. (Fig. 3c). The pathways enriched for MF mainly involved metabolism-related enzyme activity (Fig. 3d).

### Development and estimation of the prognostic model consist with eleven metabolism‑related genes

**Fig. 4:**
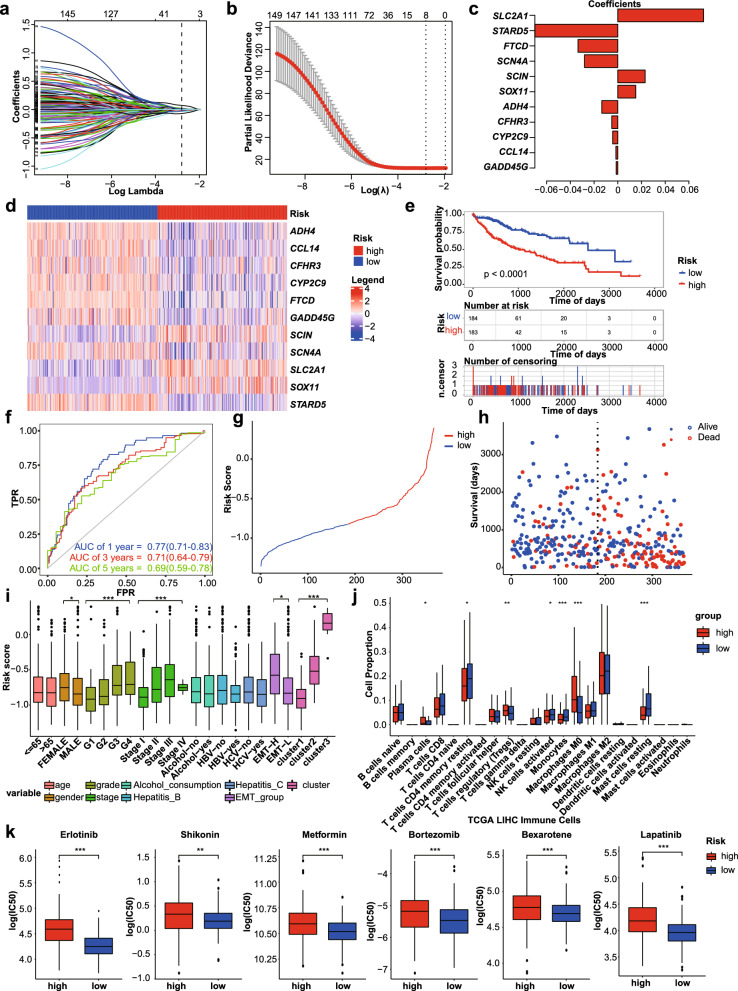
Eleven-genes-based prognostic model construction. **a** Lasso-Cox regression coefficient selection and variable screening. **b** Cross-validation in the LASSO-Cox regression model to select the tuning parameter. **c**. Display of regression coefficients corresponding to filtered variables. **d**. Heat maps of gene expression in prediction models. **e**. Validation of KM curves for models. **f**. Validation of ROC curves. **g**. Distribution of risk scores for all samples. **h**. Scatter plots of survival time for all patients. **i**. Correlation of risk scores with various clinical characteristics. **j**. Analysis of the difference in the proportion of immune-infiltrating cells between high- and low-risk groups. **k** Prediction of chemotherapy resistance in patients from high and low-risk groups. * p < 0.05, ** *p* < 0.01, and *** *p* < 0.001

We identified 150 genes that were significantly associated with overall survival (P < 0.01) by univariate Cox regression analysis from 638 DEGs (Additional file 3: Fig S3a), and top six genes showed the Kaplan–Meier curve (Additional file 3: Fig. S3b). Eleven genes were then selected as active covariates to evaluate the patients’ risk score using the Lasso Cox regression algorithm (Fig. 4a–c). The risk score was calculated using the following formula: risk score $=STARD5\times (-0.0699)+FTCD\times (-0.0335)+SCN4A\times (-0.0281)+ADH4\times (-0.0134)+CFHR3\times (-0.0050)+CYP2C9\times (-0.0043)+CCL14\times (-0.0016)+GADD45G\times (-0.0013)+SOX11\times 0.0151+SCIN\times 0.0231+SLC2A1\times 0.0723$. The above equation shows that high levels of *STARD5, FTCD, SCN4A, ADH4, CFHR3, CYP2C9, CCL14*, and *GADD45G* were prognosis-protective factors associated with low risk. However, high expression of *SOX11*, *SCIN*, and *SLC2A1* was associated with high risk. We divided the sample into high (n = 183) and low-risk groups (n = 184) using the median risk score as the cut-off point, and the expression of 11 genes was shown in the heat map (Fig. 4d). Kaplan–Meier curve showed that the high-risk group had a lower survival rate than low-risk group (Fig. 4e, g, h) (*p* < 0.0001). The prognostic model area under the curve (AUC) values of the time-dependent ROC curve were 0.77, 0.71, and 0.69 for 1 year, 3 year, and 5 year survival, respectively, which demonstrated that the multi-gene signature had better prognostic performance in predicting patient outcomes (Fig. 4f). Next, we performed risk stratification of patients according to their clinical characteristics. The results showed significant differences in risk scores between grade, stage, and EMT subgroups (Fig. 4i), which supported the accuracy of our risk model. Additionally, compared to the low-risk group, the number of plasma cells, Tregs, and macrophages M0 was significantly higher, while T cells CD4 + memory resting, NK cells activated, monocytes, mast cells resting were significantly lower (Fig. 4j). We also analyzed response to the drugs Erlotinib, Shikonin, Metformin, Bortezomib, Metformin, and Lapatinib, and found that the high-risk group was more likely to show resistance to the drugs (Fig. 4k). Hence, our risk model might be useful in predicting the response to immunotherapy and chemotherapy in patients with HCC.

### External prognostic and diagnostic validation of the eleven-genes-based prognostic model

**Fig. 5:**
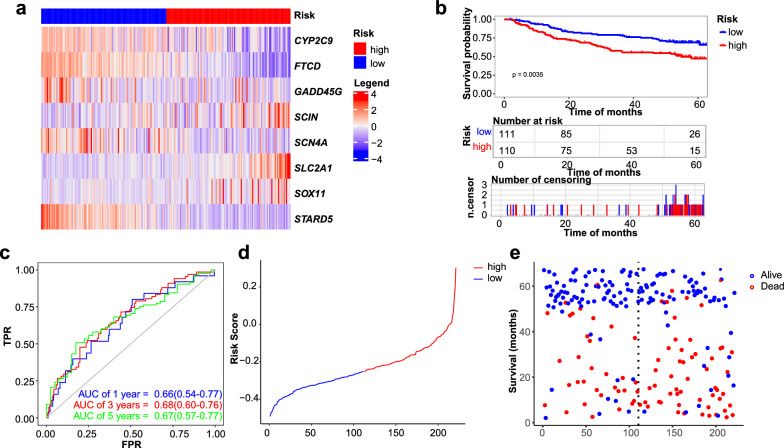
External validation of the efficacy for the risk model using GSE14520. **a**. Heat maps of gene expression in prediction models. **b**. Validation of KM curves for models. **c**. Validation of ROC curves. **d**. Distribution of risk scores for all samples. **e**. Scatter plots of survival times for all patients

We downloaded the GSE14520 dataset as a validation set, from which 221 samples with survival data were extracted. Risk scores were calculated according to the model of each patient. The expression of some genes in the prognostic model was shown in the heat map (Fig. 5a). Kaplan–Meier survival analysis showed that the high-risk group had poorer OS rate than low-risk group (*p* = 0.0035) (Fig. 5b, d, e). The prognostic model AUC values of the time-dependent ROC curve were 0.66, 0.68, and 0.67 for 1 year, 3 ear, and 5 year survival, respectively, indicating good predictive power of the prediction model (Fig. 5c). In addition, we also used the LIRI-JP database from ICGC data portal as a validation set, which also validated the stability of the model (Additional file 4: Fig. S4a–e).

### Validation of expression level of eleven genes in HCC tissues

**Fig. 6:**
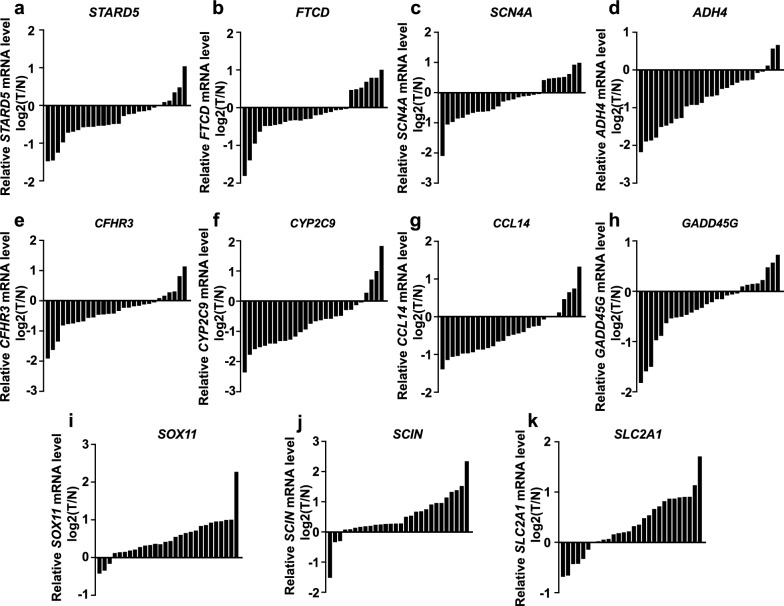
The qRT-PCR results of 11genes. **a**–**h**. *STARD5, FTCD, SCN4A, ADH4, CFHR3, CYP2C9, CCL14*, and *GADD45G* were weakly expressed in HCC tissues. **i**–**k**. *SOX11, SCIN,* and *SLC2A1* were upregulated in HCC tissues. *N* adjacent normal tissue, *T* tumor tissue

To validate whether the expression of the 11 genes in HCC tissues was consistent with our model, we determined their expression in tumors and adjacent normal tissues of 28 HCC patients by qRT-PCR. The results suggested that the mRNA levels of *STARD5, FTCD, SCN4A, ADH4, CFHR3, CYP2C9, CCL14*, and *GADD45G* were downregulated (Fig. 6a–h), and *SOX11, SCIN,* and *SLC2A1* were overexpressed in HCC tissues (Fig. 6i–k). Furthermore, the representative protein expression of nine genes in HCC and normal liver tissues was retrieved from the Human Protein Atlas (https://www.proteinatlas.org), of which two genes were not found. Consistent with the qRT-PCR results, the protein level of S*TARD5, FTCD, ADH4, CYP2C9, CCL14*, and *GADD45G* were low in HCC tissues (Additional file 5: Fig. S5a–f). SOX11, SCIN, and SLC2A1 were overexpressed in HCC tissues (Additional file 5: Fig. S5g–i).

### Stard5 down-regulation is associated with poor prognosis of HCC

**Fig. 7:**
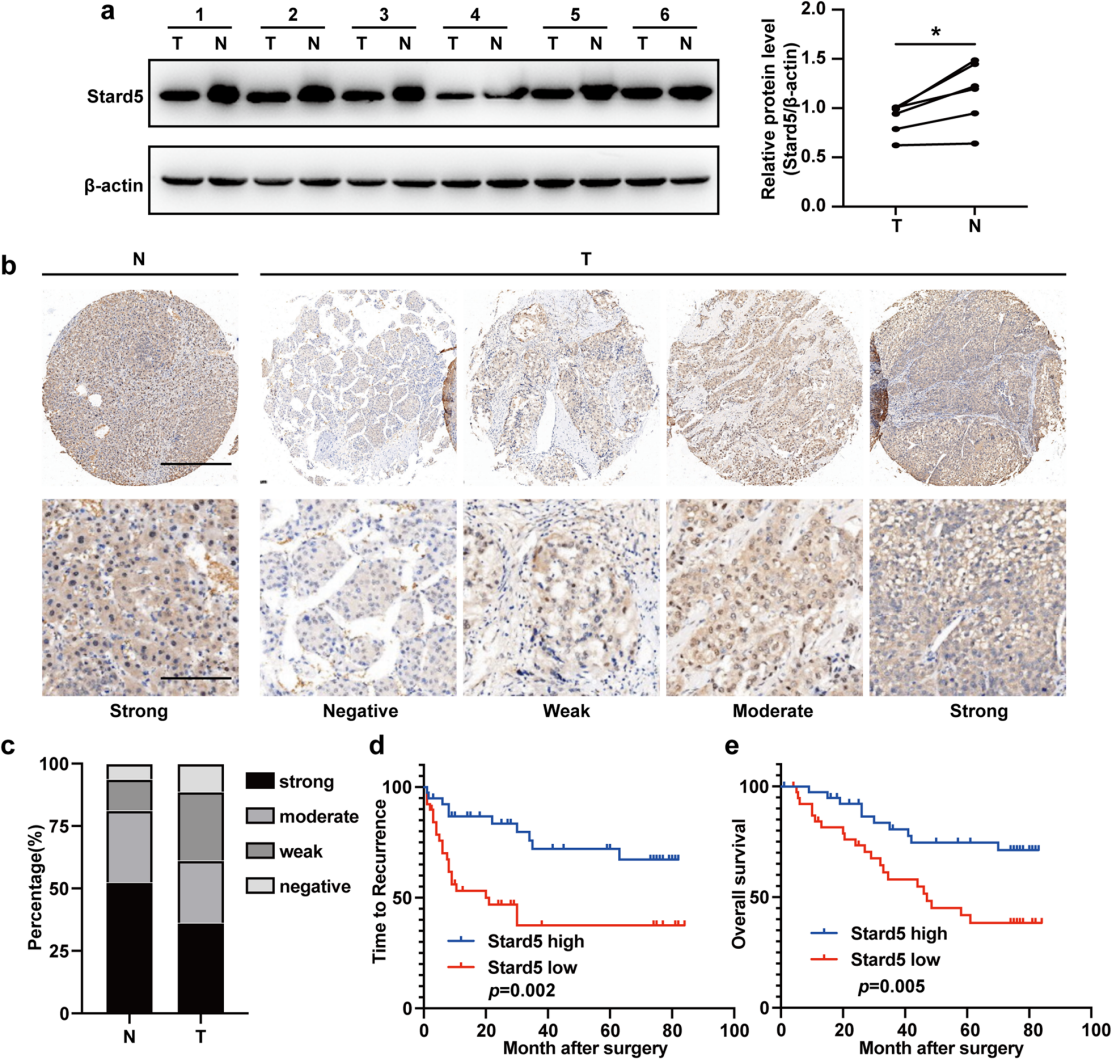
Stard5 downregulation is associated with poor prognosis of HCC. **a**. Six pairs of tumor and para-tumor tissues were randomly selected for western blot analysis (left panel). The relative intensity of Stard5 protein was normalized to β-actin (right panel). **b**, **c**. Representative IHC image percentage of Stard5 expression in HCC tumor (T) and para-tumor tissues (N). Scale bar, 500 μm or 125 μm. **d**, **e** Time to recurrence and overall survival of HCC patients in the high and low group were estimated using the Kaplan–Meier method. * p < 0.05

Currently few studies are available on Stard5 in cancer, with one paper showing that hypermethylation of the *STARD5* in clear cell renal cell carcinoma is significantly associated with poor prognosis [17]. Our prognostic model indicated that Stard5 deficiency in HCC was associated with poor prognosis, and we observed that *STARD5* mRNA was markedly decreased in 82% (23/28) of HCC tumors compared to the corresponding adjacent normal liver tissue (Fig. 6a). Western blot analysis of six randomly selected pairs of HCC samples showed that Stard5 expression was significantly reduced in tumors (Fig. 7a). Furthermore, in 80 HCC patients, Stard5 expression was scored as moderately positive in 25%, while 36.25% were strongly positive, compared to 28.75% moderately positive and 52.5% strongly positive in adjacent normal liver tissue (Fig. 7b, c). Next, patients were divided into low (negative and weakly positive) and high (moderately and strongly positive) expression groups based on Stard5 expression in the tumor tissue. Kaplan–Meier survival analysis showed that low Stard5 expression was associated with significantly poorer TTR (Fig. 7d) and shorter OS (Fig. 7e) than high Stard5 expression. Together, these data suggest that reduced Stard5 expression in tumor tissues may be an important indicator of poor prognosis in HCC.

### Stard5 deficiency promotes EMT and metastasis in HCC

**Fig. 8:**
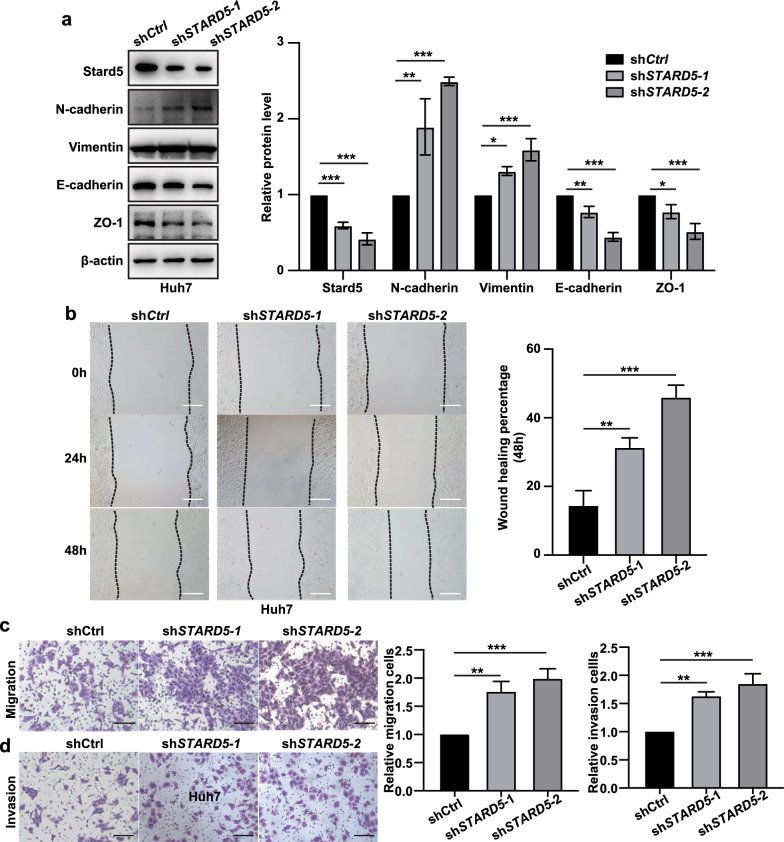
Knockdown of Stard5 promotes migration and invasion of HCC cells. **a**. Representative image of western blot showing the effect of stard5 downregulation on the EMT pathway. **b**–**d**. Wound-healing and transwell assays were used to determine the migration (**b, c**) and invasion (**d**) abilities of the referred HCC stable cells. Scale bar, 50 μm. Quantification of the relative area or relative number of cells (**b-d**, right panel) was performed by ImageJ. **p < 0.01, ***p < 0.001

**Fig. 9:**
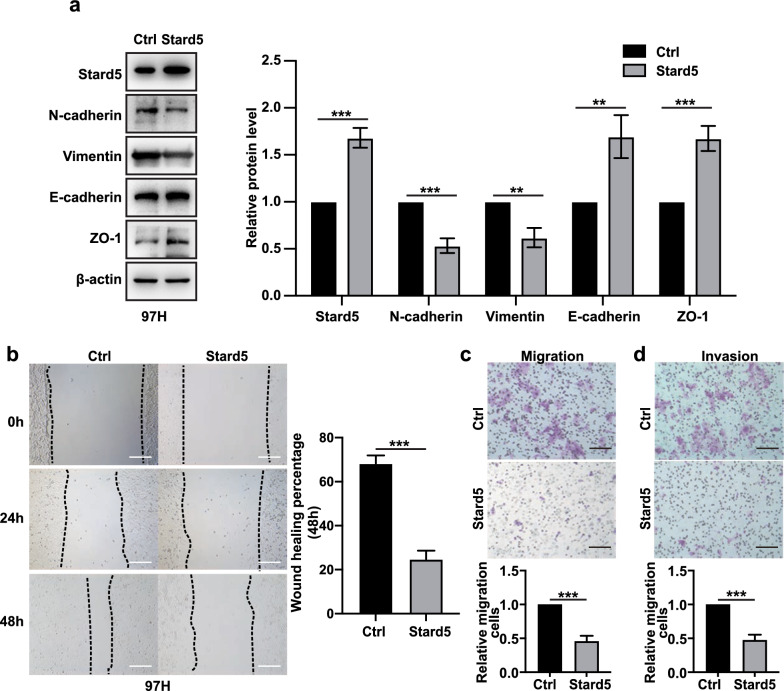
Stard5 overexpression inhibits migration and invasion of HCC cells. **a**. Representative image of western blot showing the effect of stard5 overexpression on the EMT pathway. **b**–**d**. Wound-healing and transwell assays were used to determine the migration (**b, c**) and invasion (**d**) abilities of the referred HCC stable cells. Scale bar, 50 μm. Quantification of the relative area or relative number of cells (**b**–**d**, right panel) was performed by ImageJ. ***p < 0.001

**Fig. 10:**
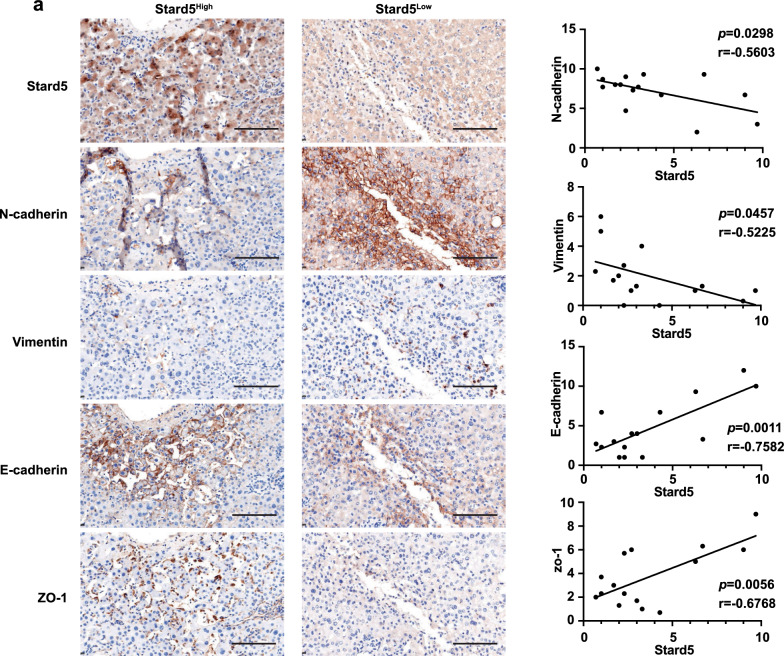
The correlation between Stard5 and EMT in HCC tissues. (**a**) Representative IHC images of Stard5, N-cadherin, Vimentin, E-cadherin and zo-1 expression in HCC patient paraffin section. Pearson’s correlation of IHC score was calculated in 15 HCC tissues

Stard5 was derived from our EMT-related prognostic model, so we first examined the impact of Stard5 on EMT. Western blot analysis showed that knockdown of Stard5 increased the expression of mesenchymal markers (N-cadherin and Vimentin) and decreased the expression of epithelial markers (E-cadherin and zo-1) in Huh 7 cells (Fig. 8a). EMT is an important step before cell invasion and migration, and we subsequently explored the role of stard5 in the invasion and migration of Huh 7 cells. In the wound-healing and transwell assays, the migration and invasion abilities decreased in sh*STARD5* cell line (Fig. 8b–d). Furthermore, overexpression of Stard5 in Huh 7 cells decreased the levels of Vimentin and N-cadherin and increased those of E-cadherin and zo-1 (Fig. 9a). Upregulation of Stard5 inhibited cell invasion and migration (Fig. 9b–d). We then detected N-cadherin, Vimentin, E-cadherin, zo-1 as well as Stard5 protein in 15 human HCC samples. Stard5 expression was scored and classified into stard5 low- and high- expression groups according to the median. The expression of mesenchymal markers (N-cadherin and Vimentin) was negatively associated with Stard5 expression, while epithelial markers (E-cadherin and zo-1) were positively associated with Stard5 expression (Fig. 10a). These data suggest that Stard5 deficiency induces EMT, resulting in HCC metastasis.

## Discussion

In our study, we constructed a risk model selected from multiple metabolic pathways based on differences between the high and low EMT groups in HCC, which contained 11 metabolism-related genes. The high-risk group had poorer prognosis than the low-risk group. The high-risk group was positively associated with Tregs and negatively associated with CD4 + T cells, NK cells. In addition, the high-risk group was more likely to develop drug resistance. These revealed that our model might support the prediction of patients’ response to chemotherapy and immunotherapy and provide a reference for individualized therapy for patients with HCC. More importantly, we demonstrated for the first time that Stard5 expression was positively correlated with survival time of HCC patients and negatively correlated with EMT, providing a precise therapeutic target.

EMT is a key cellular process that transforms polarized epithelial cells into a mesenchymal phenotype with increased cell motility. In cancer, EMT allows malignant cells to separate from the primary tumor and spread into the circulation, a critical process for invasion and metastasis [18]. Altogether, EMT is an inevitable state that occurs prior to invasion and metastasis and could be used for predicting cancer progression and prognosis. Therefore, it is logical to group patients according to their EMT enrichment scores, which may eliminate the impact of many confounders on prognosis.

We can reasonably speculate that the energy requirements of a cell switching between motion and resting states must be altered, ultimately leading to metabolic reprogramming. In fact, recent evidence suggests that the link between EMT and metabolism is reciprocal, and that altered metabolism can drive EMT in some cases. Multiple metabolic pathways, involving in glucose metabolism, lipid metabolism, amino acids metabolism, mitochondrial biosynthesis, and many other events, are simultaneously altered in tumor progression and metastasis [14]. Currently prognostic models of HCC are mostly constructed from mono-metabolic part, rather than multiple metabolic parts. In contrast, we screened and constructed risk model by comparing metabolic pathways of carbohydrates, LIPID, NUCLEOTIDE, TCA, ENERG, VITAMIN, and AMINOACID between EMT subgroups. It will be more reliable in predicting prognosis, in view of its closer proximity to the molecular biological level of cancer cells prior to invasion. In our study, there were differences in metabolism between the EMT groups, most notably in terms of energy and lipids. We classified patients with HCC into three molecular subtypes based on genes of differential metabolic pathways. Prognostic differences existed among these three subtypes, and cluster 1 had the best prognosis. The KEGG analysis showed a major focus on cell polarity, extracellular matrix, and lipid metabolic pathways. Thus, targeting specific metabolic enzymes has the potential to reverse EMT and ultimately limit cancer metastasis.

In recent years, molecular prognostic markers have received increasing attention for predicting the survival of HCC [19, 20]. Compared to single gene marker, multi-gene models have the advantage of higher predictive accuracy and more individualized results. Based on the DEGs among 3 clusters, a prognostic risk model including 11 genes (*STARD5, FTCD, SCN4A, ADH4, CFHR3, CYP2C9, CCL14, GADD45G, SOX11, SCIN*, and *SLC2A1*) was developed using univariate Cox and LASSO-Cox regression. External databases validated this risk model as valid and stable in predicting the prognosis of patients with HCC. SLC2A1, also named GLUT1, have been widely confirmed overexpressing in HCC and promoting metastasis [21]. SOX11 was also significantly upregulated in HCC [22]. FTCD, ADH4, CFHR3, CYP2C9, CCL14, GADD45G was down-regulated in HCC [23–28]. These papers supported the high accuracy of our prognostic model. Among them, FTCD and CFHR3 have been reported to play a suppressive role in the invasion and migration of HCC [23, 29]. No studies have shown a link between the remaining molecules and EMT in HCC. In addition, there are no reports on the expression of SCN4A or SCIN in HCC. We demonstrate for the first time that they were risk factors for HCC and were associated with EMT, which contributes to a better understanding of the molecular mechanisms of HCC progression. These molecules may be crucial triggers in controlling HCC metastasis.

Then, tumor microenvironment of patients was analyzed in the two groups. High risk group tended to be more immunosuppressed, with higher Tregs, and fewer CD8 + T, CD4 + T, and NK cells. In addition, patients in the high-risk group were more resistant to chemotherapy. These results imply that the risk model can help predict the effectiveness of immunotherapy and chemotherapy. Targeting these metabolic genes may improve the response of patients to treatment and provide new ideas for personalized medicine.

Stard5 became the focus of our attention, which had hardly been studied in cancers. Stard5, a lipid-binding protein, has a conserved steroidogenic acute regulatory protein-related lipid transfer domain [30]. It is involved in the regulation of cholesterol homeostasis in vivo by binding and transporting cholesterol and other sterol-derived molecules to the liver [31]. In hepatocytes, Stard5 reduces lipid accumulation, suggesting that Stard5 dysregulation may play an important role in fatty liver disease [31]. Mutations in the *STARD* gene may lead to autoimmune diseases or cancer [32]. Additionally, Mulford et al. showed that knockdown of Stard5 expression resulted in reduced sensitivity of lung cancer cells to etoposide [33]. In the present study, we demonstrated for the first time that Stard5 was down regulated in HCC tissue, and low Stard5 expression suggested poor prognosis. Stard5 deficiency contributed to the invasion and migration in HCC cell lines, while overexpression of Stard5 showed the opposite effect. The protein expression of EMT pathway was associated with Stard5 expression. These data suggest that Stard5 was a protective factor in patients with HCC.

Studies have found that endoplasmic reticulum (ER) stress increases Stard5 expression in mouse hepatocytes, and that Stard5 plays a key role in ER cholesterol homeostasis during ER stress [31].

When tumor cells experience ER stress in response to intrinsic and extrinsic changes, a network of adaptive signals, known as the unfolded protein response (UPR), will be evoked to restore protein homeostasis. UPR hyperactivation has been demonstrated to regulate cell survival, angiogenesis, inflammation, invasion, and metastasis [34]. Tumors exploit UPR signaling to promote EMT [35]. Therefore, we speculate that when ER stress occurs, Stard5 may transport excess cholesterol from the ER to the Golgi and then to the efflux pathway during the UPR, preventing excessive cholesterol accumulation in the ER, restoring ER homeostasis, and promoting apoptosis. When stard5 deficiency, ER stress induces cholesterol imbalance, the UPR may be hyperactive and unfolded proteins activate ER-resident sensors, which in turn promotes the EMT. However, it remains to be experimentally verified. Targeting stard5 directly during EMT with concomitant metabolic reprogramming may offer a prospective direction for targeting therapy.

Some limitations remain in our study, the function of stard5 in inhibiting EMT still needs to be further explored. The role of the other 10 genes in HCC remains to be studied in vitro and in vivo. Furthermore, normal tissues require the same metabolic pathways for their survival and proliferation. This implies that targeting tumor metabolism faces a series of challenges.

## Conclusions

In conclusion, we constructed a multigene prognostic model associated with EMT and metabolic reprogramming that can effectively predict the prognosis of HCC patients and determine whether patients will be able to respond to chemotherapy or immunotherapy. For the first time, we show that Stard5 can act as a tumor suppressor to inhibit EMT as well as tumor progression. Our findings provide a reference for studying the interaction between EMT and metabolic reprogramming and inhibiting tumor metastasis through therapeutic approaches targeting key metabolic molecules. This provides the basis for the development of precision medicine for targeted metabolism in the treatment of aggressive tumors.

### Supplementary Information

**Additional file 1: Figure S1.** Clinical characteristics and common signature gene analysis of metabolic pathways. (a). Heat map of the distribution of clinical characteristics in EMT subgroups. (b). Upset map of genes shared by metabolic pathways.

**Additional file 2: Figure S2.** (a) Heat map of differentially expressed genes by subtype.

**Additional file 3: Figure S3.** Cox regression analysis of 638 DEGs. (a). Forest plots for bulk univariate cox regression analysis, showing only the top 20 genes. (b). KM curves for bulk univariate cox regression analysis, showing only the top 6 genes.

**Additional file 4: Figure S4.** External validation of the efficacy for the risk model using LIRI-JP database. (a). Heat maps of gene expression in prediction models. (b). Validation of KM curves for models. (c). Validation of ROC curves. (d). Distribution of risk scores for all samples. (e). Scatter plots of survival times for all patients.

**Additional file 5: Figure S5.** Immunohistochemical staining of genes in risk model. (a-f) STARD5, FTCD, ADH4, CYP2C9, CCL14, and GADD45G were expressed at low levels in HCC tissues. (g-i) SOX11, SCIN, and SLC2A1 were upregulated in HCC tissues.

## Data Availability

The datasets generated and analyzed during the current study are available in TCGA (http://cancergenome.nih.gov/abouttcga) and GEO (https://www.ncbi.nlm.nih.gov/geo).

## Abbreviations

HCC: Hepatocellular carcinoma
TCGA: The Cancer Genome Atlas
EMT: Epithelial-mesenchymal transition
DEGs: Differentially expressed genes
GEO: Gene expression omnibus
KEGG: Kyoto Encyclopedia of Genes and Genomes
GO: Gene Ontology term
BP: Biological processes
CC: Cellular component
MF: Molecular function
AUC: Area under the curve
ROC: Receiver operating characteristic
ER: Endoplasmic reticulum
UPR: Unfolded protein response
