# Supplementary figures and images for: Eleven metabolism‑related genes composed of Stard5 predict prognosis and contribute to EMT phenotype in HCC
Source: Cancer Cell Int. 2023 Nov 17;23:277. doi: 10.1186/s12935-023-03097-0 (PMC10656919; doi:10.1186/s12935-023-03097-0)

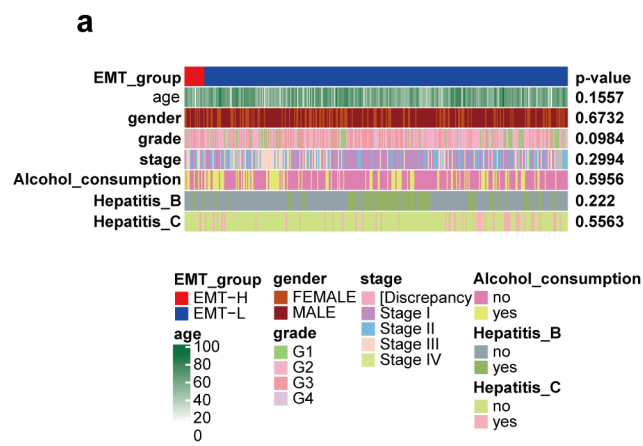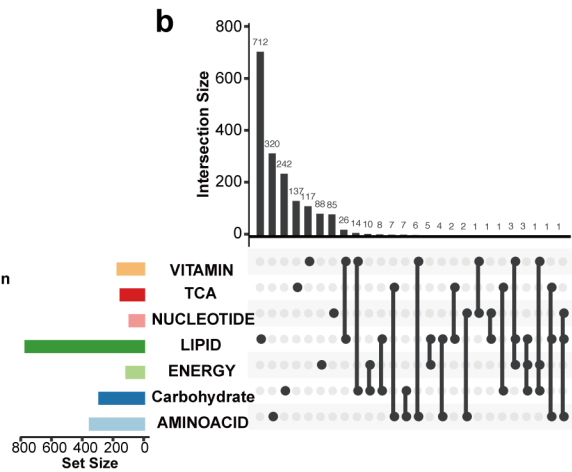

Supplement: Supplementary file 1 — Additional file 1: Figure S1. Clinical characteristics and common signature gene analysis of metabolic pathways. (a). Heat map of the distribution of clinical characteristics in EMT subgroups. (b). Upset map of genes shared by metabolic pathways. [file 12935_2023_3097_MOESM1_ESM.pdf]

**a**

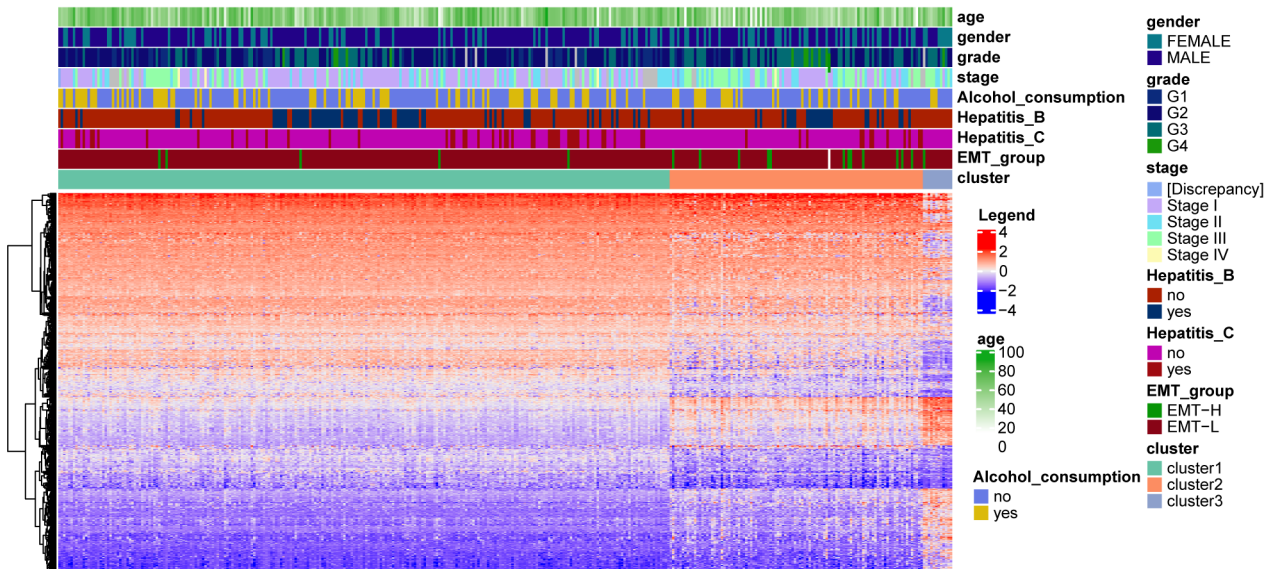

Supplement: Supplementary file 2 — Additional file 2: Figure S2. (a) Heat map of differentially expressed genes by subtype. [file 12935_2023_3097_MOESM2_ESM.pdf]

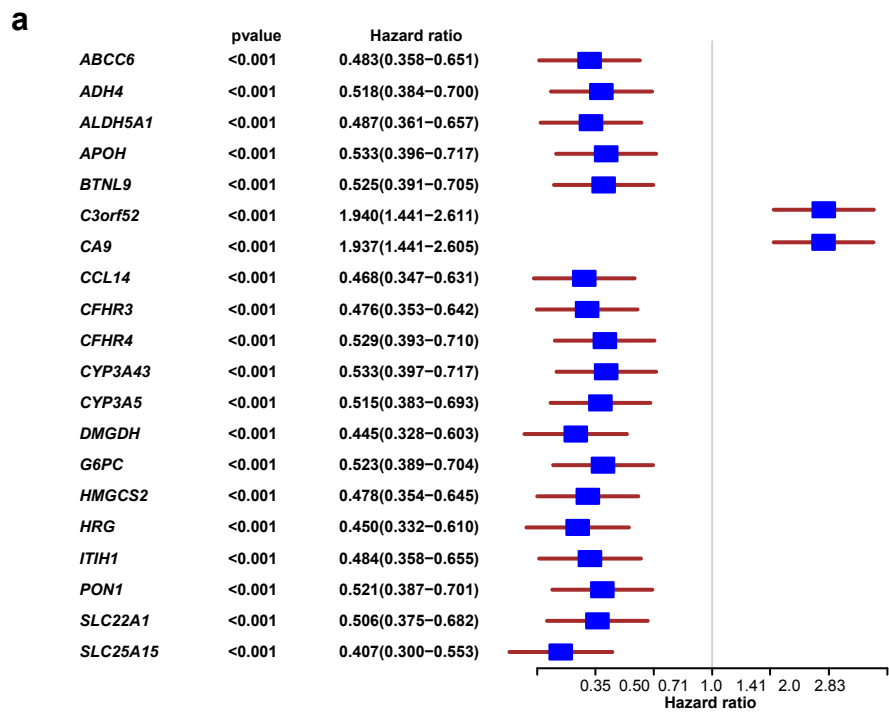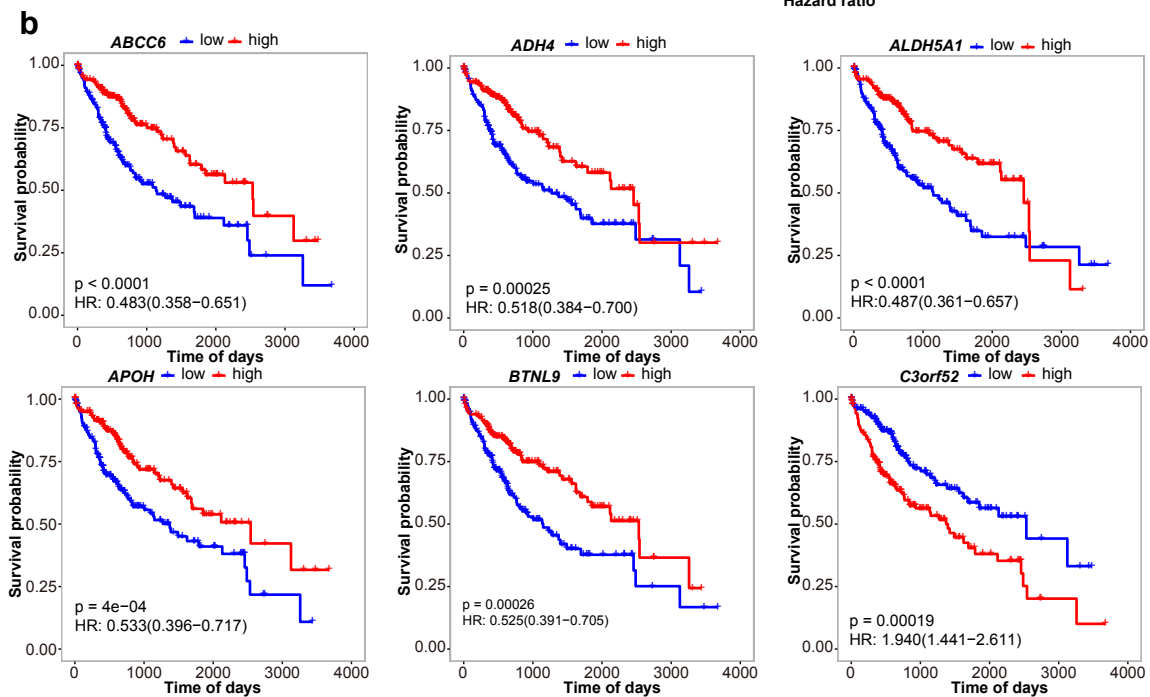

Supplement: Supplementary file 3 — Additional file 3: Figure S3. Cox regression analysis of 638 DEGs. (a). Forest plots for bulk univariate cox regression analysis, showing only the top 20 genes. (b). KM curves for bulk univariate cox regression analysis, showing only the top 6 genes. [file 12935_2023_3097_MOESM3_ESM.pdf]

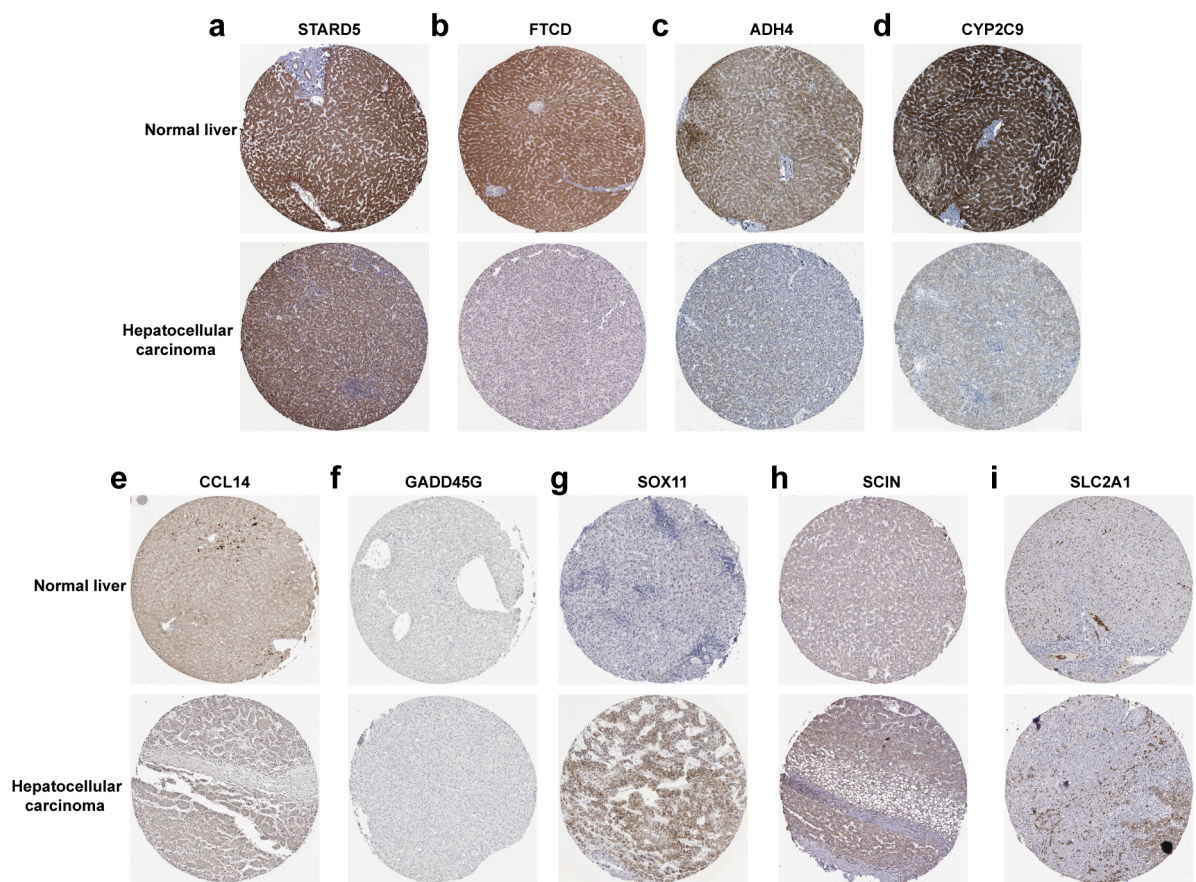

Supplement: Supplementary file 5 — Additional file 5: Figure S5. Immunohistochemical staining of genes in risk model. (a-f) STARD5, FTCD, ADH4, CYP2C9, CCL14, and GADD45G were expressed at low levels in HCC tissues. (g-i) SOX11, SCIN, and SLC2A1 were upregulated in HCC tissues. [file 12935_2023_3097_MOESM5_ESM.pdf]
